# Underdosed and Underappreciated: Why Japanese Community-Acquired Pneumonia Outcomes With Ampicillin-Sulbactam May Not Translate Globally

**DOI:** 10.1093/ofid/ofaf348

**Published:** 2025-06-11

**Authors:** Hayato Mitaka, Karrine Brade, Alyssa Y Castillo

**Affiliations:** Division of Infectious Diseases, University of Colorado School of Medicine, Aurora, Colorado, USA; Antimicrobial Stewardship Program, University of Colorado Hospital, Aurora, Colorado, USA; Antimicrobial Stewardship Program, University of Colorado Hospital, Aurora, Colorado, USA; Department of Pharmacy, University of Colorado Hospital, Aurora, Colorado, USA; Division of Infectious Diseases, University of Colorado School of Medicine, Aurora, Colorado, USA; Antimicrobial Stewardship Program, University of Colorado Hospital, Aurora, Colorado, USA

To the  Editor—We read with great interest the recent article by Yamamoto and colleagues [1], which demonstrated higher mortality for older adults hospitalized with community-acquired pneumonia (CAP) who were treated with ampicillin/sulbactam (SAM) as compared with ceftriaxone. However, key historical and epidemiologic context specific to Japan must be considered before extrapolating these findings to patients in current-day Japan and other countries.

First and foremost, SAM has been systematically underdosed in Japan. Since its approval in 1994, the SAM package insert in Japan has stipulated that this antibiotic be used at 6 g daily, divided into 2 doses (ie, 3 g every 12 hours). This outdated drug information does not reflect the pharmacokinetic and pharmacodynamic profile of SAM and is inconsistent with international guidelines [2] and package inserts from other countries. The mean half-life for SAM is 1 to 1.3 hours, and 75% to 85% is excreted in the urine as unchanged drug. Considering the peak lung concentrations from previous literature, doses <3 g every 6 hours in patients with normal renal function would not achieve the target attainment goal of >50% time above the minimum inhibitory concentration for many organisms [3]. Therefore unsurprising, in a small study comparing 4-times-daily vs twice-daily dosing of SAM in Japanese older adults with pneumonia, 4 times daily was more effective in patients with gram-negative pneumonia [4]. The dosing regimen of 3 g every 6 hours became available in Japan only after 2012, when the drug information was updated and allowed for the use of higher and more frequent doses in “serious infections,” which were not clearly defined. Moreover, it was not until 2015 that a study was published examining the efficacy and safety of this dose in Japanese patients with CAP [5]. This is perhaps collateral evidence of the slow uptake of every-6-hour dosing, which remains far from the norm in Japan. Because the period studied by Yamamoto and colleagues was 2010 to 2023, the SAM group received less frequent dosage than recommended internationally, at a minimum from 2010 to 2012, but likely for many patients thereafter. While Japanese pneumonia guidelines exist and recommend dosing every 6 to 8 hours, the document is available online free of charge only to members of the Japanese Respiratory Society. Since the majority of small- to medium-sized hospitals in Japan do not have access to an infectious disease specialist (physician and/or pharmacist) who would advocate for appropriate antibiotic usage, it is plausible that many hospitalized patients with CAP still receive every-12-hour dosing as it remains recommended in the package insert. In contrast, ceftriaxone dosage in Japan is 1 to 2 g daily divided into 1 to 2 doses (eg, 1–2 g every 24 hours, 1 g every 12 hours), consistent with international guidelines [2]. There is no information on antibiotic dosing in the present study, which prompts the following question: Could the worse outcomes associated with SAM based on Japanese real-world data simply reflect the inappropriate dosing of the antibiotic throughout the country?

Second, the resistance pattern of CAP pathogens in Japan differs from that in the United States. Specifically, BLNAR (β-lactamase nonproducing ampicillin resistant) *Haemophilus influenzae* is prevalent in Japan, and 40% to 45% of *H influenzae* isolates are considered resistant to SAM and amoxicillin/clavulanate [6]; in contrast, in the United States, only 1.9% of *H influenzae* isolates are resistant to these drugs [7]. This might also account for the slightly higher mortality with SAM in Japan, limiting the generalizability to other countries.

In conclusion, while the slightly higher mortality detected in the present study among older adults with CAP who received SAM vs ceftriaxone based on real-world data in Japan may accurately reflect the current situation in Japan, the applicability to other countries may be limited. Third-generation cephalosporin use is consistently identified as a risk factor for extended-spectrum β-lactamase (ESBL) development [8], which has prompted several academic institutions in the United States to shift from ceftriaxone to SAM as their first-line therapy for CAP. Notably, reducing ESBL selection pressure by reducing unnecessary use of third-generation cephalosporins is also a national focus in response to antimicrobial resistance [9] in Japan, where approximately 30% of inpatient *Escherichia coli* isolates are assumed to be ESBL based on national surveillance data [10]. For the reasons detailed here, we propose that this study should not be a barrier to further studying opportunities to utilize appropriately dosed SAM in Japan to help achieve this antimicrobial resistance aim [9]. Before judging one drug as worse than the other, infectious disease specialists should continue to advocate for appropriate dosing of SAM based on its pharmacokinetic and pharmacodynamic profile, including pushing for the revision of the outdated package insert in Japan.

## Notes

***Author contributions.*** All authors have seen and approved the manuscript and contributed significantly to this work.
